# A large-scale viability assessment of the potato cryobank at the International Potato Center (CIP)

**DOI:** 10.1007/s11627-017-9846-1

**Published:** 2017-08-24

**Authors:** Rainer Vollmer, Rosalva Villagaray, José Cárdenas, Mario Castro, Oswaldo Chávez, Noelle L. Anglin, David Ellis

**Affiliations:** International Potato Center, Lima, Peru

## Introduction

The International Potato Center (CIP) in Lima, Peru, maintains the global in-trust collection of cultivated potato. The material from this collection is distributed worldwide on request for research, breeding, and education under the auspices of the International Treaty for Plant Genetic Resources for Food and Agriculture (ITPGRFA, http://www.fao.org/plant-treaty/en/) (FAO 2009). The 4388 accessions in this collection are maintained *in vitro* to ensure that once cleaned of viruses, the germplasm remains virus free and can move freely across international borders without introducing disease(s) to other countries. While *in vitro* maintenance safeguards the material from infection, it requires a large number of resources to maintain an *in vitro* collection of this size. One way to reduce the cost of maintenance while still conserving the germplasm is to back up the collection in a cryobank. In fact, cryopreserved material could be the primary storage for some accessions that are genetically unique, but rarely requested. The shoot tips can be thawed from liquid nitrogen and regenerated into a plantlet when needed for research or distribution purposes.

Cryopreservation protocols applicable to dozens of crops have been developed during the last two decades and many plant genetic research institutes have cryobanks that conserve hundreds or up to thousands of accessions (Pritchard 2016). However, throughput rates for large collections, quality standards, number of stored samples and repetitions, minimum viability criteria, inter-vial variation, backup storage strategies, and effective linkage with other genebank programs (for example, genetic identity testing) vary widely between species and cryobanks. These variables are often dependent on resources, staffing, and other priorities in the genebanks. At CIP, clear and strict quality standards were implemented in 2013, defining many of the characteristics described above (Vollmer *et al.* 2016). Despite this, the development of protocols applicable to large-scale cryopreservation of diverse collections, even within a single crop, can be a time-consuming, evolving, and a continuous process.

The pioneers of potato cryopreservation published their first protocols about 35–40 yr ago (Bajaj 1978, 1981; Grout and Henshaw 1978; Towill 1981, 1983, 1984). Although the initial methods permitted successful cryopreservation of some potato genotypes, the development and adaptation of these protocols to high-throughput cryopreservation of diverse potato collections needed further refinement and simplification for routine use. Researchers at the Leibniz Institute DSMZ (German Collection of Microorganisms and Cell Cultures) refined the protocols in the mid-1990s, so a cryopreservation method could be successfully applied to hundreds of potato genotypes (Schäfer-Menuhr *et al.* 1994, 1997). Recently, researchers have published other promising methods for potato cryopreservation, such as the D and V cryoplate. In these methods, shoot tips are “stuck” on special aluminum plates with uniform wells using sodium alginate to facilitate uniform sample handling, reduced risk of damage, and reduced loss of shoot tips (Yamamoto *et al.* 2015; Valle *et al.* 2017).

At CIP, potato cryopreservation was initiated in the late 1990s (Golmirzaie and Panta 2000) by a protocol developed for *Musa* species (Panis *et al.* 2005) and the protocol has been continuously modified and improved (Panta *et al.* 2015; Vollmer *et al.* 2016). In 2013, CIP’s cryobank established a clear and strict quality management system (QMS), started to build a cryobank for the future with defined quality standards (Vollmer *et al.* 2016), and began actively adding on average 400 accessions per year into the cryobank. This paper describes the current status of CIP’s cryobank, analysis by potato species/subspecies (taxonomy based on Hawkes 1990), ploidy level, and country of origin to determine factors that affect the success rate, and the progress made over the last few yr. It also includes the results of a large-scale viability reassessment of 857 potato accessions, conserved for 1–2 yr in liquid nitrogen, and an assessment of the influence of the human factor on the survival and recovery rates of potato accessions cryopreserved with the droplet vitrification method (Sakai *et al.* 1990).

## Materials and Methods

### Propagation of plant material

*In vitro* plantlets were subcultured into GA_7_ Magenta™ boxes (Química Service SRL, Lima, Peru) every 3 wk onto semi-solid-modified MS medium (Murashige and Skoog 1962) (Medium Type: MSP09, Caisson Laboratories, East Smithfield, PA) supplemented with 25 g L^−1^ of sucrose (S8501, Sigma-Aldrich®, St. Louis, MO) and 3.0 g L^−1^ of Phytagel™ (P8169, Sigma-Aldrich®). The pH was adjusted to 5.6 ± 0.02 with NaOH (2 M) and HCl (2 M). The culture media was autoclaved at 121°C for 20 min. Plants were incubated at 18–22°C with a 16-h photoperiod and light intensity of 80–100 μmol m^−2^ s^−1^ provided by cool day light fluorescent tubes (TL-D 36W/865, Philips Lightning S.A., Miraflores, Lima, Perú). For the final transfer, terminal buds were subcultured on the same culture medium in 2.5 cm deep Petri dishes (70 buds per Petri dish) and incubated for 2–3 wk at 6–8°C at low light intensity (15–25 μmol m^−2^ s^−1^), with a photoperiod of 16 h.

### Excision of shoot tips

Shoot tips were excised from 2- to 3-wk-old cold-acclimated *in vitro* grown plantlets (6–8°C, see description of final transfer in previous section). Excised shoot tips contained 3–4 leaf primordia with a length of 0.8–1.2 mm and a width of 0.4–0.7 mm. A total of 130–150 shoot tips were excised for each accession.

### Cryoprotection

Shoot tips were treated for 20 min with loading solution (LS) [liquid-modified MS medium (Caisson Laboratories, MSP09) containing 2.0 M glycerol (G5516, Sigma-Aldrich®) and 0.4 M sucrose, adjusted to pH 5.8] at 20–24°C, followed by treatment with plant vitrification solution 2 (PVS2) [liquid-modified MS medium containing 3.28 M glycerol, 2.42 M ethylene glycol (324558, Sigma-Aldrich®), 1.9 M dimethylsulfoxide (DMSO) (D4540, Sigma-Aldrich®), and 0.4 M sucrose, adjusted to pH 5.8] for 50 min on ice (0°C).

### Freezing in liquid nitrogen

Just prior to the end of the PVS2 treatment, 10 shoot tips were placed in a 10-μL pre-chilled (0°C) droplet of PVS2 on a sterile aluminum foil strip (5 × 20 mm) (Boardwalk® BWK 7136, Essendant Co., Deerfield, IL) and quickly plunged into liquid nitrogen (LN) (− 196°C) immediately after a 50-min exposure to PVS2. The frozen aluminum foil strip was transferred into a 1.8-mL cryovial (internal thread Nunc™, Thermo Fisher Scientific™, Waltham, MA) under LN so that each cryovial contained 10 shoot tips. For routine cryopreservation, 130–150 shoot tips (13–15 cryotubes) per accession were processed. After a minimum of 24 h in LN, a sample of 30 shoot tips (three vials) were thawed to obtain an initial survival and recovery rate.

### Thawing

Aluminum foil strips with 10 frozen shoot tips were quickly thawed by removing the foil strip from the cryovial and submerging it directly into rewarming solution (RS) (liquid-modified MS medium containing 1.2 M sucrose, adjusted to pH 5.8). The shoot tips were rewarmed in RS for 20 min at 20–24°C.

### Recovery

Shoot tips were recovered in 1.5 cm deep Petri dishes on semi-solid recovery medium consisting of a modified MS medium, supplemented with 0.4 mg L^−1^ kinetin (K3378, Sigma-Aldrich®), 0.1 mg L^−1^ gibberellic acid (G7645, Sigma-Aldrich®), 20 mL L^−1^ coconut water (C5915, Sigma-Aldrich®), 0.3 M sucrose, and 2.8 g L^−1^ Phytagel™ (P8169, Sigma-Aldrich®). The pH was adjusted to 5.6 ± 0.02 with NaOH (2 M) and HCl (2 M). The culture medium was autoclaved for 20 min at 121°C. Filter-sterilized kinetin, gibberellic acid, and coconut water stock solutions were added to the culture medium after autoclaving. Three pieces of filter paper (grade 2, brand: Whatman®, Sigma-Aldrich®), each containing the 10 shoot tips from a single vial were placed in each Petri dish. Shoot tips were incubated for 3 d in darkness at 18–22°C by wrapping the Petri dishes with aluminum foil, followed by 3 d on modified MS culture media of the same composition, but with decreased sucrose concentrations of 0.1 and 0.2 M (both in darkness). After 9 d in darkness, shoot tips were removed from the filter paper and placed directly onto culture medium with the same composition as the recovery medium, but containing 0.07 M sucrose, and incubated for 4 d under diffuse light at 18–22°C with a 16-h photoperiod (covering the top of the Petri dishes with a sheet of aluminum foil), followed by normal light conditions (80–100 μmol m^−2^ s^−1^). After 30 d, surviving and recovered shoot tips were transferred individually into 13 × 100 mm test tubes, containing the same medium (0.07 M sucrose). Recovered samples without root formation were cut slightly at the base before transferring to the test tubes.

### Viability assessment

Viability was assessed 30 and 60 d after thawing. After 60 d, a shoot was classified as recovered, if it had developed into a complete and morphologically normal looking *in vitro* plant (elongated stem, functional apex, leaves, and roots). If the samples did not develop into complete *in vitro* plants, the samples were recorded as survived, but not recovered. Shoot tips that developed into deformed plants showed only leaf formation or had signs of hyper-hydration (vitrification) were classified as survived, but not recovered.

### Transfer to the cryobank and safety backup tank

Based on the observed recovery rate after 60 d, previously determined QMS standards governed whether an accession was transferred to the cryobank, if a second attempt at cryopreservation (cryorun) was required, or if the current cryorun was discarded. For accessions that had a recovery rate of 30% or higher, one single cryorun was stored in the cryobank with two vials set aside in a separate cryotank for a safety backup. For accessions that showed a recovery rate of 20–30% in the first cryorun, an additional independent second cryorun was performed and both runs were stored if both runs had recovery rates > 20%. Those cryoruns with 20–30% recovery rates had four vials stored as a safety backup. Accessions with less than 20% recovery rates were discarded and not transferred to the cryobank.

### Ploidy determination

The ploidy levels of the potato accessions were determined using flow cytometry utilizing a BD Accuri™ C6 flow cytometer (BD Biosciences, San Jose, CA). For sample preparation, 50–60 mg of young leaf tissue was cut in a Petri dish containing 250 μL of LB01 buffer. LB01 buffer was prepared as described by Doležel *et al.* (1989). Additionally, 250 μL of LB01 buffer was added to the suspension and incubated for 2 min at 20–24°C, followed by filtering through a 50-μm CellTrics® filter (Sysmex Corp, Lincolnshire, IL). Staining of nuclei was done with 50 μg mL^−1^ propidium iodide (Calbiochem, San Diego, CA), staining of double-stranded RNA was avoided by adding 50 μg mL^−1^ of RNase (R6513, Sigma-Aldrich®). After an additional incubation period of 2 min in darkness, samples were analyzed by flow cytometry at medium run speed (with 1000 and 10,000 events for the FL2-H and FSC-H threshold, respectively). Each run had a duration of 2 min with a minimum of 400 events per G0/G1 peak. Results were interpreted by comparison with reference standards of known potato ploidy levels. Manual chromosome counts of root tips were performed as described by Orillo and Bonierbale (2009) when required.

### Data analysis

**Figure 1.**
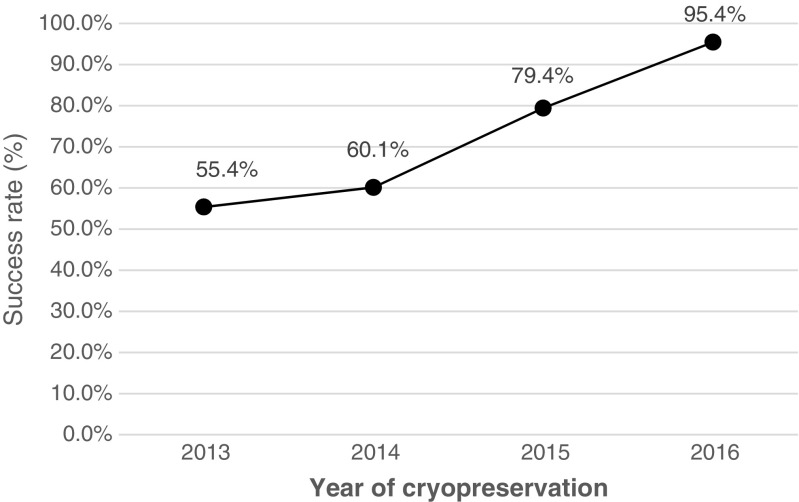
Success rate (%) of potato cryopreservation at CIP (2013 to 2016). The success rate (%) is expressed as the relationship between successfully cryopreserved and totally processed accessions. An accession is considered as successfully cryopreserved if it had a minimum recovery rate of 30% or higher (one single cryopreservation attempt) or 20–30% (two independent cryopreservation attempts).

Data was analyzed with a one-way ANOVA test (*p* < 0.05) and the Tukey multiple comparison test (*p* < 0.05) using the MINITAB 17.3.1 and EXCEL 2016 software. Accessions that did not fulfill the minimum reassessment criteria (8 accessions), or had pending viability reassessment results (4 accessions) were excluded from all analyses (Table 1). The *Solanum* ×*ajanhuiri, S.* ×*curtilobum,* and *S.* ×*juzepczukii* species were excluded from the statistical analyses for the inter-specific comparison, as these groups contained only 2–11 accessions. All species were included (sample size per group 109 to 575) for the comparison of survival and recovery rates between years 2013–2016. For the evaluation of the effect of ploidy level on survival and recovery rates, 1327 of 1533 potato accessions with confirmed ploidy levels (2×, 3×, 4×) were included. However, the pentaploid species (*S.* ×*curtilobum*) was excluded from statistical analysis because of a low sample number (*n* = 2). The variance of survival and recovery rates of the accessions based on country of origin was analyzed and countries with a low sample size were excluded from the analyses, e.g., Bangladesh (*n* = 2), Bhutan (*n* = 1), Costa Rica (*n* = 1), Guatemala (*n* = 8), Mexico (*n* = 6), Netherlands (*n* = 1), and Russia (*n* = 9) (Table 4). The success rate for cryopreservation was expressed as a ratio between successfully cryopreserved and totally processed accessions (Fig. 1).

**Table 1.** The number of originally processed accessions, number of accessions that did not pass the viability reassessment check, and number of accessions with pending viability reassessment

Description	Year of cryopreservation				
	2013	2014	2015	2016	Total
Number of cryopreserved accessions (originally)	113	415	442	575	1545
Number of accessions that did not pass the viability reassessment	4	4	0	–^c^	8
Number of accessions with pending viability reassessment^a^	0	1	3	–^c^	4
Total^b^	109	410	439	575	1533

^a^The viability reassessment of 4 accessions, cryopreserved in 2014/2015 accessions is still pending

^b^Total = number of cryopreserved accessions - number of accessions that did not pass the viability reassessment - number of accessions with pending viability reassessment

^c^The viability of the accessions cryopreserved in 2016 will be reassessed in 2017/2018 (after 1–2 yr in LN)

### Viability reassessment

**Figure 2.**
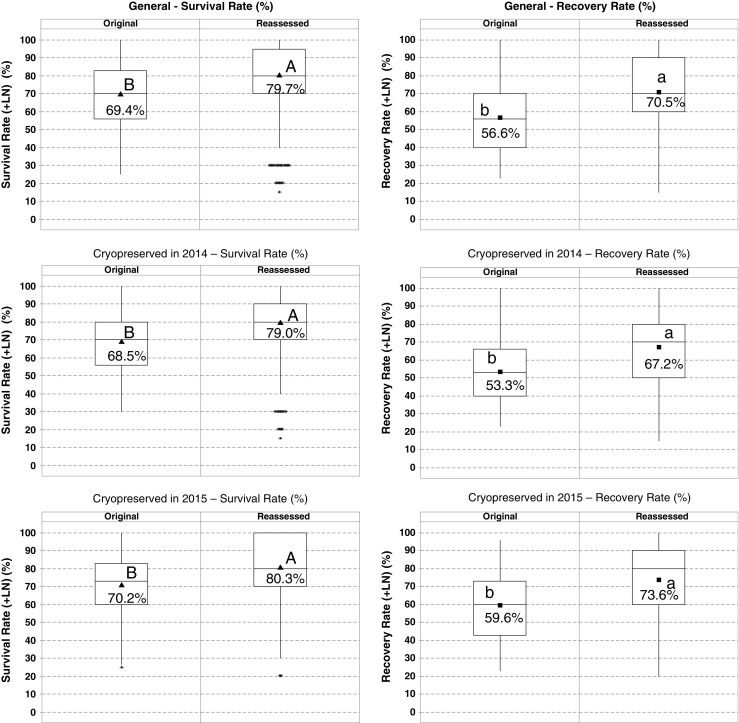
Box plot graphs of original and reassessed survival and recovery rates of 849 accessions that were successfully cryopreserved at CIP’s cryobank in the years 2014–2015. Original and reassessed data are based on sample sizes of 30 and 10 shoot tips, respectively. *Black triangle:* mean of survival rate; *black square:* mean of recovery rate; *asterisks:* outlier values. *Different letters* indicate significant differences for the two-sample *t* test (*p* < 0.05). Statistical differences between survival rates are indicated with *capital letters*, and differences between recovery rates are indicated with *lowercase letters*. Mean values of survival and recovery rates are indicated within the box plot graphs.

The viability of the accessions cryopreserved in 2014 and 2015 was assessed after a period of 1–2 yr in LN. The recovery rates from this reassessment were compared with the average recovery rates of the three original cryovials (30 shoot tips) thawed ~ 24 h after freezing for the baseline viability (referred to as the “original viability”). The reassessment consisted of removing an additional cryopreserved vial for each accession from the cryobank, thawing, and recovering as described above with the seemingly minor change that the recovery cycle, 30 to 60 d post-thawing, was performed in 25 × 150 mm instead of 13 × 100 mm test tubes. Two 25 × 150 mm test tubes per accession were used, placing a maximum of five shoot tips per test tube. Only samples that showed survival or recovery after 30 d were transferred to the test tubes (Fig. 2).

In total, the viability of 857 potato accessions from 2014 to 2015 was reassessed (Table 1). Data was analyzed with box plots and a two-sample *t* test (*p* < 0.05) (Fig. 2). Additionally, the recovery rates of the vial with the central (median-valued vial) and maximum value of the original viability (based on 10 shoot tips, respectively) were compared with the recovery rate of the reassessed vial (based on 10 shoot tips) (Table 5).

**Figure 3.**
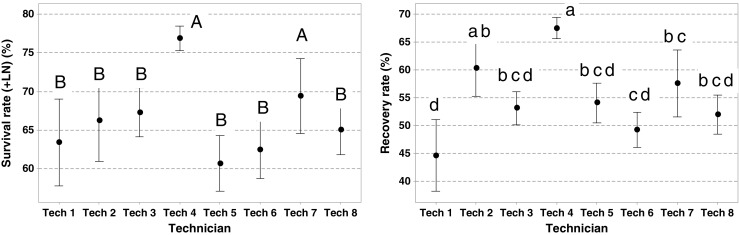
The survival and recovery rates of 979 potato accessions, routinely cryopreserved by eight different technicians (years 2015 to 2016). Tech 7 is the senior technical staff member, who trained all the other staff members (Tech 1–6 and Tech 8). *Filled circle:* median values. *Different letters* indicate significant differences for the Tukey test for the means (*p* < 0.05). Statistical differences between survival rates are indicated with *capital letters*, and differences between recovery rates are indicated with *lowercase letters*.

### Influence of the human factor on cryopreservation

The survival and recovery rates of cryopreserved potato based on the eight different technicians conducting the procedure were compared using a group of 979 accessions cryopreserved in 2015 and 2016. Technician performance was measured based on routinely obtained survival and recovery rates of cryopreserved potatoes. Technician 7 was the most experienced technical staff member, who trained the other technicians (Tech 1–6 and Tech 8) (Fig. 3). Sample sizes ranged from 45 to 377 accessions per technician.

**Figure 4.**
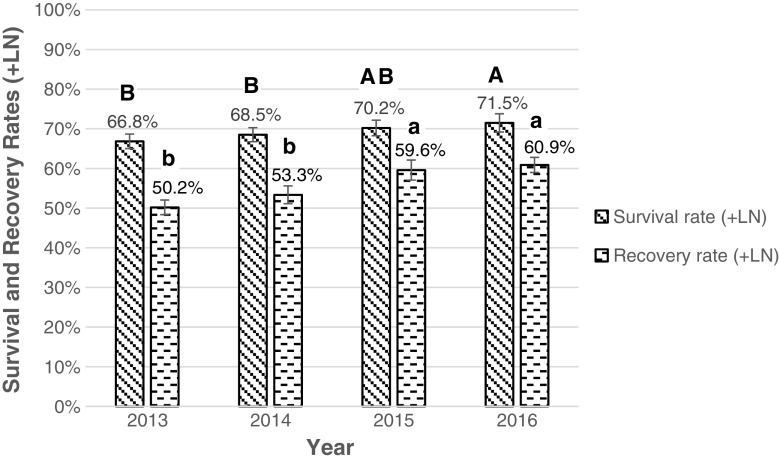
Average survival and recovery rates (+LN) of 1533 potato accessions that were routinely cryopreserved at CIP between 2013 and 2016. *Different letters* indicate significant differences based on the Tukey multiple comparison test (*p* < 0.05). Statistical differences between survival rates are indicated with *capital letters*, and differences between recovery rates are indicated with *lowercase letters*. Average survival and recovery rates per year are indicated above the graphs. Standard errors are indicated by *bars*.

## Results

### Routine cryopreservation

In 2016, a higher number of potato accessions (575 accessions) were successfully cryopreserved compared to previous years (2013: 113 accessions; 2014: 415 accessions; 2015: 442 accessions) (Table 1). The average recovery rates (+LN) of the accessions cryopreserved in 2016 (60.9%) and 2015 (59.6%) were significantly higher than in the two prior years 2014 (53.3%) and 2013 (50.2%). Also, the average survival rate of the 2016 cryopreserved accessions (71.5%) was higher compared to 2014 (68.4%) and 2013 (66.8%) (Fig. 4).

The cryopreserved material belonged primarily to the accessions of tetraploid taxa *Solanum tuberosum* subsp. *andigenum* (1126), followed by *S. stenotomum* subsp. *stenotomum* (122), *S. tuberosum* subsp. *tuberosum* (74), *S. phureja* (55), *S. chaucha* (48), and *S. stenotomum* subsp. *goniocalyx* (44). Comparisons among the material successfully cryopreserved were made to evaluate whether specific attributes such as taxonomic classification, ploidy level, or country of origin influenced success rates. Based on comparisons using the Tukey multiple comparison test (*p* < 0.05), *S. tuberosum* subsp. *tuberosum* showed a significantly higher average recovery rate of 64.2% than the taxa *S. phureja* (53.1%), *S. stenotomum* subsp. *goniocalyx* (52.8%), and *S. stenotomum* subsp. *stenotomum* (48.9%). *Solanum tuberosum* subsp. *andigenum* showed a significantly higher average recovery rate of 58.9% than *S. stenotomum* subsp. *stenotomum* (48.9%), but no significant differences were observed when compared with the other analyzed species and subspecies (Table 2).

**Table 2.** Survival and recovery rates (±standard error, SE) of 1533 potato accessions cryopreserved at CIP in the years 2013 to 2016. The accessions belong to seven species (based on Hawkes 1990) and four different ploidy levels (2×, 3×, 4×, and 5×). All accessions were cryopreserved with the PVS2-droplet cryopreservation method (LS 20 min, PVS2 50 min on ice, RS 20 min). Survival and complete plant recovery was assessed 60 d after thawing

Species/subspecies	Number of cryobanked accessions	Average survival rate (+LN) (% ± SE)^(1)^	Average recovery rate (+LN) (% ± SE)^(1)^
*Solanum tuberosum* subsp. *tuberosum*	74	75.7 ± 2.3 A	64.2 ± 2.3 a
*Solanum tuberosum* subsp. *andigenum*	1126	70.4 ± 0.5 AB	58.9 ± 0.5 ab
*Solanum* ×*chaucha*	48	67.4 ± 2.5 AB	56.2 ± 2.9 abc
*Solanum* ×*juzepczukii* ^(2)^	11	65.9 ± 4.0^(2)^	54.8 ± 5.5^(2)^
*Solanum phureja*	55	66.0 ± 2.3 B	53.1 ± 2.5 bc
*Solanum stenotomum* subsp. *stenotomum*	122	66.9 ± 1.5 B	48.9 ± 1.5 c
*Solanum stenotomum* subsp. *goniocalyx*	44	68.5 ± 2.3 AB	52.8 ± 2.6 bc
*Solanum* ×*ajanhuiri* ^(2)^	9	66.9 ± 6.7^(2)^	51.1 ± 5.7^(2)^
*Solanum* ×*curtilobum* ^(2)^	2	38.5 ± 2.5^(2)^	32.0 ± 2.0^(2)^
*Solanum* sp. (hybrids)	42	69.3 ± 2.3 AB	57.7 ± 2.2 abc
Mean		70.0 ± 0.4	57.7 ± 0.5

^(1)^ *Different letters* indicate significant differences based on the Tukey multiple comparison test (*p* < 0.05). Statistical differences between survival rates are indicated with *capital letters*, and differences between recovery rates are indicated with *lowercase letters*

^(2)^ *Solanum* ×*juzepczukii, Solanum* ×*ajanhuiri,* and *Solanum* ×*curtilobum* were excluded from the statistical analysis, as they contained a low sample number of 2–11 accessions per species

A Tukey test was conducted to determine if ploidy level influenced the cryopreservation success rate. Tetraploid (4×) accessions had a significantly higher average recovery rate (58.9%) than diploid (2×) accessions (50.7%). No significant differences were observed in either survival or recovery rates between triploid (3×) and tetraploid (4×) accessions (Table 3). Geographic origin, defined as the country where the accession was collected, was also assessed to determine if this had an effect on cryopreservation success. Accessions from Chile had a significantly higher average recovery rate (65.1%) than accessions from Peru (57.1%). No statistically significant differences were observed between the average recovery rate of the accessions coming from Chile, Colombia, Ecuador, and Venezuela (Table 4).

**Table 3.** Survival and recovery rates (±standard error, SE) of 1329 cryopreserved potato accessions classified by its ploidy level (2×, 3×, 4×, and 5×). Ploidy level was determined by flow cytometry (BD Accuri C6). Accessions with missing ploidy data were excluded from the analysis. Accessions were cryopreserved with the PVS2-droplet cryopreservation method (LS 20 min, PVS2 50 min on ice, RS 20 min). Survival and complete plant recovery was assessed 60 d after thawing

Ploidy level	Number of cryopreserved accessions	Average survival rate (+LN) (% ± SE)^(1)^	Average recovery rate (+LN) (% ± SE)^(1)^
2×	200	66.9 ± 1.2 B	50.7 ± 1.2 b
3×	95	67.6 ± 1.5 AB	54.6 ± 1.9 ab
4×	1032	70.6 ± 0.5 A	58.9 ± 0.6 a
5×^(2)^	2^(2)^	38.5 ± 2.5^(2)^	32.0 ± 2.0^(2)^

^(1)^ *Different letters* indicate significant differences based on the Tukey multiple comparison test (*p* < 0.05). Statistical differences between survival rates are indicated with *capital letters*, and differences between recovery rates are indicated with *lowercase letters*

^(2)^The pentaploid accessions were excluded from the statistical analysis, because of a low sample number (*n* = 2)

**Table 4.** Survival and recovery rates (±standard error) of 1533 cryopreserved potato accessions from 2013 to 2016, classified per country of origin. All accessions were cryopreserved with the PVS2-droplet cryopreservation method (LS 20 min, PVS2 50 min on ice, RS 20 min). Survival and complete plant recovery were assessed 60 d after thawing

Country of origin	Number of cryopreserved accessions	Average post +LN survival rate (% ± SE)^(1)^	Average post +LN recovery rate (% ± SE)^(1)^
Argentina	27	68.0 ± 2.9 AB	57.5 ± 3.1 ab
Bangladesh^(2)^	2	69.5 ± 6.5	66.0 ± 10.0
Bolivia	230	71.1 ± 1.1 AB	57.7 ± 1.2 b
Bhutan^(2)^	1	70.0	56.0
Chile	70	76.1 ± 2.3 A	65.1 ± 2.3 a
Colombia	100	71.8 ± 1.7 AB	59.5 ± 1.9 ab
Costa Rica^(2)^	1	66.0	56.0
Ecuador	65	67.0 ± 1.9 B	56.0 ± 2.3 ab
Guatemala^(2)^	8	66.9 ± 5.4	59.4 ± 4.0
Mexico^(2)^	6	76.8 ± 6.6	65.7 ± 7.9
Netherlands^(2)^	1	76.0	56.0
Peru	990	69.4 ± 0.5 B	57.1 ± 0.6 b
Russia^(2)^	9	69.0 ± 5.7	60.1 ± 7.2
Venezuela	23	67.8 ± 3.8 AB	58.6 ± 3.3 ab

^(1)^ *Different letters* indicate significant differences based on the Tukey multiple comparison test (*p* < 0.05). Statistical differences between survival rates are indicated with *capital letters*, and differences between recovery rates are indicated with *lowercase letters*

^(2)^Countries with low sample size of < 9 accessions per country were excluded from the statistical analysis

An evaluation of the influence of staff technicians and their experience on the success of the cryopreservation process was also analyzed (Fig. 3). Technician (Tech) 4 showed a significantly higher average cryopreservation recovery rate of 67.5% compared to the remaining Techs (44.6–57.6%). Tech 4 had 2 yr of experience in the cryopreservation process and showed a significantly higher recovery rate compared to a technician with more than 20 yr of experience (Tech 7). No significant differences were observed between the senior staff Tech 7 (69.4%) and Tech 4 (76.9%) in terms of the cryopreservation survival rates, but, both showed a significantly higher average cryopreservation survival rate than the other technicians. The average cryopreservation recovery and survival rates of Tech 4, Tech 3, and Tech 8 were based on larger sample sizes of 377, 171, and 128 accessions, respectively.

### Viability reassessment

Periodic viability reassessment of cryopreserved material is crucial to confirm the stability of viability over time, as is the case for seed collections. Viability reassessment after 1–2 yr is important to establish the functional QMS for cryopreservation and to identify potential problems or accessions with suboptimal viability at an early stage.

It was interesting to observe that the survival and recovery rates of the viability reassessment were significantly higher (*p* < 0.05) than the original viability data. The mean values of the reassessed survival and recovery rates were 10.3 and 13.9% higher, respectively, than the original data (Fig. 2). The difference between the reassessed average survival and recovery rates of the 2015 material was 6.7%, which is 4% lower than the original data (10.6%). A similar tendency was observed for the 2014 material (Δ reassessment 11.8%; Δ original 15.2%). Four of the 857 reassessed accessions (< 0.5%) showed signs of contamination (two vials were tested), and thus, the accessions were discarded from the cryobank.

For 552 of 849 accessions (65%), the reassessed vial showed a higher recovery rate than the median-valued vial of the original assessment (Table 5).

**Table 5.** Comparison between the vials with the highest and middle recovery rates of the three routinely assessed vials and the recovery rates of the reassessed vial (849 accessions). In this case, recovery rates of the routinely evaluated and reassessed vials were both based on a sample size of 10 shoot tips. The table shows the number of accessions (total 849 accessions); the reassessment showed lower, equal, or higher recovery rates compared to one single vial (median or maximum value) of the original routine data

Description	Comparison of recovery rates	Number of accessions
Compared to the middle-valued vials^a^ of the routine recovery rate	Routine > reassessment	175
	Routine = reassessment	122
	Routine < reassessment	552
Compared to the highest-valued vials^b^ of the routine recovery rate	Routine > reassessment	336
	Routine = reassessment	153
	Routine < reassessment	360

^a^The middle-valued vial (or median-valued vial) of each accession is that single vial of the three routinely assessed vials that had the central value of recovery

^b^The highest-valued vial of each accession is that single vial of the three routinely assessed vials that had the highest value of recovery

## Discussion

During 2016, an increase in the post-thawing survival and recovery rates of cryopreserved potato accessions was observed. Specifically, in *S. tuberosum* subsp. *tuberosum*, a noteworthy increase in the average recovery rate from 59.0 to 64.2% was obtained (Vollmer *et al.* 2016). The continuous improvement of the survival, recovery (+LN), and yearly throughput rates in CIP’s cryobank during the years 2013–2016 can be attributed to an increase in the technical abilities and experience of the research staff as no major changes in the protocol were made. Interestingly, the Institute of Plant Genetics and Crop Plant Research (IPK) in Germany reported the opposite effect in the cryopreservation of their potato genebank, with a decreasing trend for recovery of cryopreserved accessions with time (Keller *et al.* 2014).

Results of new and promising cryopreservation methods, like the dehydration (D)- and vitrification (V)-plate protocols, show high recovery rates of 70–93.3% (13 accessions; D cryoplate) and 93.3–100% (16 accessions; V cryoplate) (Yamamoto *et al.* 2015; Valle *et al.* 2017), but the assessment of these methods on a larger scale (for example, > 500 randomly selected accessions) has not yet been performed. It is also important to highlight that CIP’s potato cryobank exclusively conserves landraces, while other collections contain higher quantities of improved potato varieties or breeding material, which tend to be less variable and have a narrower genetic base than landraces. The intra-specific recovery rates reported in the present work are also based on hundreds of accessions and, therefore, are difficult to compare to more specific protocols using a very limited number of accessions. Practicality of the cryomethod, yearly throughput rate per person, and strict viability assessment criteria (complete plant recovery) are all important factors that need to be considered for any large-scale cryobank operation.

In CIP’s cryobank, higher average recovery rates (+LN) were observed for the *S. tuberosum* subsp. *tuberosum* and subsp. *andigenum* (64.2 and 58.9%), *S. chaucha* (56.2%), and *S.* ×*juzepczukii* (54.8%), while the diploid species showed lower average recovery rates ranging from 48.9 to 53.1% (Table 2). A direct relationship between ploidy level and cryoresponse could be analyzed, but needs to be further investigated. *S. stenotomum* subsp. *stenotomum* showed a high differential of > 18% between the average survival and recovery rates. The pentaploid *S. curtilobum* had a low average recovery rate of 32.0%, but this is based on a very small sample size (2 accessions) and it cannot be concluded that it is a characteristic of this particular ploidy level. The optimization of the cryopreservation protocols for the diploid *S. stenotomum* subsp. *stenotomum* and the pentaploid *Solanum* ×*curtilobum* could be considered for future research projects.

As 98% of the Chilean accessions belong to the tetraploid *S. tuberosum* subsp. *tuberosum*, and showed a significantly higher recovery rate than the accessions coming from other countries, there could be a correlation between the specific ploidy or genetic composition of *tuberosum* subspecies. The Chilean accessions were originally obtained from the extreme south (between the latitude of − 42.66 and 44.37° S; data not shown). However, it is important to note that 69 of the 70 cryopreserved Chilean accessions belong to a single taxon, *S. tuberosum* subsp. *tuberosum*, which as noted above had significantly higher recovery rates that may account for this seemingly geographical difference. The viability reassessment of the 2014–2015 cryopreserved accessions confirmed a minimum recovery rate of 20% for 99% of the accessions (based on one single vial). The data of the 849 successfully reassessed potato accessions showed a higher average recovery rate of 70.5% compared to the original rate of 56.6% (an average differential of 13.9%). Finally, 79% of the reassessed accessions showed an equal or higher recovery rate compared to the median-valued vials of the routine data (Table 5).

The 30–60 d post-thaw period of the 2014–2015 reassessment was done in wider 25 × 150 mm test tubes, with more plants per tube, while the 2013 material was reassessed in 13 × 100 test tubes with one plant per tube. The 2013 material (109 accessions) had an original average recovery rate of 50.3%, and for its viability reassessment, a recovery rate of 55.1% was reported (Vollmer *et al.* 2016). Based on the observation that the differential for the 2014–2015 material was nearly three times higher than for the 2013 material, it is possible that the dimensions of the test tubes used or the number of plants per test tube influenced the recovery rate. It is also possible that the increased expertise of the cryobank staff at the time of reassessment (2014/15 material) was a factor in the higher recovery rate. This hypothesis would explain the continuously increased success rate for routine cryopreservation from 2013 to 2016 (Fig. 1). Further research is required to assess the influence of the storage vessel and the technical staff’s experience on the post-thawing recovery rate in cryopreservation.

The influence of the human factor on the survival and recovery rates has also been shown. Although Tech 7 had more than 20 yr of practical experience in cryopreservation, has cryopreserved thousands of accessions, and co-developed CIP’s cryomethod, Tech 4 apparently had found a way to increase the recovery rate substantially with putatively improved excision or sample handling. The PVS2-droplet method is a multi-step process, which requires fine motor skills in many steps, like shoot tip excision, treatment of samples with LS and PVS2, forming the PVS2 droplet, placing the PVS2 droplet (with 10 shoot tips) on a small aluminum strip, and plunging it into LN. Minor changes in sample handling could have a major influence on the recovery results. The performance of Tech 4 is currently being analyzed systematically to identify the critical step or steps that have led to the increased cryopreservation process improvement.

## Conclusions

The efficiency of CIP’s cryomethod was confirmed with a high number (1533) of potato landraces belonging to seven different species. In 2016, 95% of the processed potato accessions did accomplish CIP’s minimum viability standards (≥ 20% viability). In a viability reassessment of the 2014–2015 cryopreserved accessions, 99% of the cryopreserved accessions fulfilled the minimum viability standards, confirming the robustness of the previously established quality management system. A potential for protocol improvement by conducting a systematical review of sample handling procedures has been identified, based on the outstanding performance of one specific technician.
